# An Accessible Pre-Rehabilitation Bundle for Patients Undergoing Elective Heart Valve Surgery with Limited Resources: The TIME Randomized Clinical Trial

**DOI:** 10.31083/j.rcm2411308

**Published:** 2023-11-09

**Authors:** Zeruxin Luo, Xiu Zhang, Yuqiang Wang, Wei Huang, Miao Chen, Mengxuan Yang, Pengming Yu

**Affiliations:** ^1^Department of Rehabilitation Medicine, West China Hospital, Sichuan University, 610041 Chengdu, Sichuan, China; ^2^Department of Cardiovascular Surgery, West China Hospital, Sichuan University, 610041 Chengdu, Sichuan, China

**Keywords:** elective heart valve surgery, pre-rehabilitation, postoperative pulmonary complications, medical costs

## Abstract

**Background:**

Despite gradually increasing evidence for pre-rehabilitation for heart valve surgery, it remains underused, especially in developing countries with limited resources. The study aimed to investigate the feasibility and effects of an innovative three-day pre-rehabilitation bundle for patients undergoing elective heart valve surgery.

**Methods:**

This was a single-center, assessor-blind, randomized clinical trial. A total of 165 patients were randomly assigned to either usual care (control group, n = 83) or usual care with an additional 3-day pre-rehabilitation bundle (Three-day of Inspiratory muscle training, aerobic Muscle training, and Education (TIME) group, n = 82). The main outcome of the study was the incidence of postoperative pulmonary complications (PPCs). Secondary outcomes included the feasibility of the intervention, duration of the non-invasive ventilator, length of stay, and PPCs-related medical costs on discharge.

**Results:**

Of 165 patients 53.94% were male, the mean age was 63.41 years, and PPCs were present in 26 of 82 patients in the TIME group and 44 of 83 in the control group (odds ratio (OR), 0.60; 95% CI, 0.41–0.87, *p *= 0.006). The feasibility of the pre-rehabilitation bundle was good, and no adverse events were observed. Treatment satisfaction and motivation scored on 10-point scales, were 9.1 ± 0.8 and 8.6 ± 1.4, respectively. The TIME group also had fewer additional PPCs-related medical costs compared to the control group (6.96 *vs*. 9.57 thousand CNY (1.01 *vs*. 1.39 thousand USD), *p <* 0.001).

**Conclusions:**

The three-day accessible pre-rehabilitation bundle reduces the incidence of PPCs, length of stay, and PPCs-related medical costs in patients undergoing elective valve surgery. It may provide an accessible model for the expansion of pre-rehabilitation in countries and regions with limited medical resources.

**Clinical Trial Registration:**

This trial was based on the Consolidated Standards of Reporting Trials (CONSORT) guidelines. This trial was registered in the Chinese Clinical Trial Registry (identifier ChiCTR2000039671).

## 1. Introduction

According to the “Medical Quality Report of Cardiovascular Diseases in China (2021)”, there are currently about 330 million people with cardiovascular diseases in China [1]. The aging of China’s population is creating an explosive growth in patients with valvular heart disease (VHD) [2]. Postoperative complications, especially postoperative pulmonary complications (PPCs), remain a huge burden and challenge in the perioperative management of cardiac surgery all over the world [3]. An accumulating body of studies has demonstrated that the incidence of PPCs after cardiac surgery exceeds 50% [4, 5, 6]. The emergence of PPCs is associated with a series of high-cost medical events, increased in-hospital mortality [7, 8], and reduced quality of life [9]. This poses a huge challenge to developing countries like China, which have limited medical resources.

Taking advantage of pre-operative waiting time to make physical and psychological improvements can increase the patient’s ability to cope with surgery’s physiologic stress and reduce the risk of complications after surgery [10]. The components of pre-rehabilitation recommended by current research in cardiac surgery mainly include preoperative education, exercise training and inspiratory muscle training (IMT) [11]. Generally, pre-rehabilitation programs are recommended at home or in the community for up to two weeks or more in previous studies [12]. A growing body of evidence supports that this pattern can reduce the incidence of postoperative complications, especially PPCs, improve functional capacity, and provide a more seamless transition to recovery after discharge back to the community and work [11, 13].

Although the concept of pre-rehabilitation has been widely accepted, it has not been widely promoted in clinical practice in China. There is still a gap between strong evidence and clinical practice. This is attributable to the fact that this type of program is impractical in developing countries with limited resources. In developing regions like China, where most of the population falls into the category of low and middle-income countries (LMICs), low accessibility to cardiac rehabilitation (CR) is a common phenomenon [14]. There are also unique reasons that impede access to the pre-rehabilitation intervention in China. First, current medical resources for cardiac surgery are still far from adequate in China, only 37.3% of VHD patients underwent valvular surgery [2]. This has resulted in a shortened preoperative preparation time, with most patients having less than five days of preoperative preparation time. Second, community-based rehabilitation is still not well developed, especially in the vast rural areas [15]. This makes the feasibility of home-based or community-based pre-rehabilitation impossible to guarantee for high-risk cardiac patients. Furthermore, Chinese professional physical therapists are still a scarce medical resource. A survey of physiotherapists (PTs) practicing in China showed that the majority (85.3%) of PTs currently work in public hospitals and therefore are less likely to perform pre-rehabilitation outside the hospital [16]. Finally, the out-of-hospital health insurance payment is also a dilemma for rehabilitation in China. These factors have contributed to the low accessibility of pre-rehabilitation in Chinese patients undergoing elective cardiac surgery.

Therefore, the optimal way to adapt to current conditions is to use the short preoperative waiting time during preoperative hospitalization to provide pre-rehabilitation. Few studies have provided preliminary evidence of the feasibility and effectiveness of short-term pre-rehabilitation programs in patients undergoing elective cardiac surgery such as the preoperative intensive IMT program (training for five days, twice a day) formulated by Chen *et al*. [17]. The preoperative short-term intensive IMT program can reduce the risk of postoperative PPCs in coronary artery bypass grafting (CABG) patients. Boden *et al*. [8] also demonstrated that a 30-minute preoperative physical therapy session can halve the incidence of PPCs in upper abdominal surgery patients. To our knowledge, there are still no reports of pre-rehabilitation for isolated heart valve surgery. The cardiac surgery patients in our center are predominantly those with VHD. Most of them have a long course of the disease and do not seek medical attention until symptoms arise, resulting in delayed treatment. Therefore, these patients may have a worse physical condition than those with coronary artery disease [1]. Pre-rehabilitation interventions may have greater potential benefits for this group of patients.

The single-blinded, randomized clinical trial was designed to explore the feasibility and effectiveness of the innovative pre-rehabilitation bundle, especially the incidence of PPCs and related medical costs.

## 2. Methods

### 2.1 Trial Design/Setting

The Three-day of Inspiratory muscle training, aerobic Muscle training, and Education (TIME) study was a pragmatic, assessor-blind, noninferiority, parallel-group, randomized clinical trial, conducted in a real-world Western China cardiac surgery center. The trial was registered in the Chinese Clinical Trial Registry. This trial was based on the Consolidated Standards of Reporting Trials (CONSORT) guidelines [18].

### 2.2 Study Participants

The trial was conducted at the Department of Cardiovascular Surgery, West China Hospital of Sichuan University, from August 15, 2021, to September 15, 2022. Patients with elective heart valve surgery were eligible. Trial participants signed an informed consent form as required by the Ethics Committee and in accordance with the Declaration of Helsinki. The inclusion criteria were age 18–90 years and New York Heart Association (NYHA) classifications II–III. The exclusion criteria were cardiovascular instability, receiving pre-rehabilitation intervention within eight weeks, pulmonary infection and severe atelectasis before surgery, infective endocarditis, aortic aneurysm, and aortic dissection. Written informed consent was obtained from all participants. Withdraw criteria were surgery-related complications requiring preoperative treatment, termination of surgical treatment for any reason during hospitalization, pre-operative acute heart failure onset or presence of malignant arrhythmias, and serious postoperative adverse events with non-pulmonary complications, including massive gastrointestinal bleeding, cerebrovascular accident events, low cardiac output syndrome, and cardiac arrest.

### 2.3 Randomization, Allocation Concealment, and Blinding

The allocation sequence was determined by a computer-generated blocked random number table. An independent administrator prepared an envelope and marked the patient assignment inside the envelope according to the order table. This envelope contained assignment cards wrapped in aluminum foil. The patients were randomly assigned to either the TIME group or the control group by an independent administrator using sequentially numbered sealed opaque envelopes. All those who participated in this trial could not use this form until the trial was completed.

A qualified PT (physiotherapist) performed the pre-rehabilitation program. Independent assessors were involved in data collection but not in any medical interventions. The multidisciplinary team and patients were informed of the group assignment. Blinding was maintained by the assessor and statistical expert.

### 2.4 Interventions

All participants received standardized physical and subjective assessments on admission and a general preoperative education lesson by a cardiac nurse (CN) on the day before surgery. This contains information about the surgery, expected pain management, tubes and lines used during medical procedures, and the postoperative recovery process. All patients also received a booklet with this information.

The additional comprehensive pre-rehabilitation bundle in the TIME group includes a three-day (12 training sessions/240 minutes) intensive IMT, aerobic muscle training, and PT-led education protocol before surgery. The pre-rehabilitation multidisciplinary team consists of a cardiac surgeon, CN, and PT. The primary task of the CN is to prepare a schedule to ensure that the protocol is implemented and to assist the PT in IMT and aerobic muscle training. The PT is the primary implementer of the pre-rehabilitation bundle. The cardiac surgery specialist keeps track of the patient’s safety throughout the pre-rehabilitation bundle.

IMT was performed with a tapering flow resistance device (Blue Whale™, Xeek, Xiamen, China), and the training protocol was set according to a previous study [19]. The IMT intensity is 30% of their maximal inspiratory mouth pressure (Pi-max), measured at baseline. During the training, the intensity also increased incrementally based on the Borg scale. If the scale was less than 5, the intensity was increased incrementally by 2 ${\mathrm{cmH}}_{2}$O. The duration of each session was 20 minutes and consisted of two sessions (morning and afternoon) each day. Aerobic muscle training was carried out by symptom-limited walking training in the hospital corridor. The duration of the training was 20 minutes, and the training intensity was 60% of the heart rate reserve determined by the functional capacity test (6-minute walk test at admission). All physiotherapy was closely monitored under the supervision of a multidisciplinary team, and the cardiac surgeon made the decision to discontinue or continue the pre-rehabilitation bundle based on the patient’s condition.

An additional PT-led education protocol was given after IMT for 20 minutes. The main contents of the education protocol were based on the study by Zheng *et al*. [20]. It included knowledge about PPCs, respiratory training techniques (deep diaphragm breathing, effective coughing), self-stretching exercises, encouraging preoperative physical activity reducing daytime bed rest, knowledge of early mobilization and self-directed breathing exercises during time in the intensive care unit (ICU) and cardiac ward (**Supplementary File 1**). In each education session, the PT asked the patient to repeat the education contents to ensure that the patient had mastered these techniques. In addition, the PT answered questions about the patient’s cardiac surgery and provided additional information based on the patient’s occupation and life situation. At the end of each training session, the PT also gave the assignment to the patient after each training session to allow the patient to practice the content of the educational protocol.

Patients undergoing elective heart valvular surgery received either a catheter-based intervention (transcatheter aortic valve replacement) or open-heart surgery. The surgical team (including a cardiologist, two cardiovascular surgeons, an echocardiographer, and an anesthesiologist) selected the procedure based on the guidelines for valvular surgery [21], patient preference, and other factors. All other aspects of patient care, including preoperative preparation, prophylactic antibiotic use, pain management, and general care, were determined by the CN and physicians based on routine clinical practice. From the first postoperative day, all participants received early mobilization, chest physiotherapy, and other physical therapy by the same experienced physiotherapy team in both the ICU and cardiac wards.

### 2.5 Outcome 

#### 2.5.1 Primary Outcome Measure

The primary outcome was the incidence of PPCs within 14 days after surgery, scored by independent assessors using the definition by Hulzebos *et al*. and Kroenke *et al*. [19, 22]. The severity of complications worsened with increasing grade, and clinically significant PPCs were determined by achieving 2 items of Grade 2 or 1 item of Grade 3 or 4.

#### 2.5.2 Secondary Outcome Measures

Secondary outcomes included (1) Adverse effects during testing or training, participant satisfaction, and compliance during the intervention. The relevant safety events [23, 24, 25] were recorded based on defined criteria (**Supplementary File 2**). Cardiovascular and respiratory medical-related events were the focus of attention and were recorded by the PT or CN during training sessions. Patient satisfaction and motivation were determined after the last session by a blinded assessor. Each patient was asked to complete 11 questions that included subjective satisfaction and motivation with the overall bundle of intensity, training time, satisfaction and motivation for the entire bundle, the length of training, and the organization [26] (**Supplementary File 3**). (2) Length of hospital stay and ventilator support were also evaluated by a blinded assessor in the medical record system. (3) PPCs-related medical costs items were based on written and electronic medical records [27]. The audited items included: ward bed (intensive care unit and surgical general ward), medical consultation costs, nursing expenses, electrocardiogram monitoring fees, ventilation support costs, oxygen therapy costs, chest imaging costs, blood as well as sputum testing costs, antibiotic costs, and the cost of the pre-rehabilitation bundle (about 600 CNY (87.20 USD)) [27, 28, 29, 30]. All medical therapies during the patient’s hospitalization were decided by the attending physician, who was blinded to the patient’s allocation. Medical program fees were derived from a single center’s pricing standards for the West China Hospital of Sichuan University.

### 2.6 Sample Sizes

Sample sizes were calculated based on the primary outcome event of the study population. The pre-trial investigation of the incidence of PPCs after cardiac surgery was 58%. Other literature has published a 50% relative risk reduction in the incidence of PPCs in the cardiac surgery population after performing preoperative physiotherapy [31]. We expected a 50% relative risk reduction with the pre-rehabilitation bundle. Bilateral α = 0.05 with 90% power. A significant difference of *p* < 0.05 was calculated using PASS 15.0.13 software (NCSS, LLC. Kaysville, UT, USA). A 20% calculation for lost visits and refusals required at least 72 study patients in each group.

### 2.7 Statistical Analyses

Main analyses for all outcomes were on intention-to-treat basis. The data were tested for normality using the Kolmogorov-Smirnov test. If missing values were present, multiple Imputation of missing data was performed (R x64 4.0.4 R Foundation for Statistical Computing, Vienna, Austria) and subsequent sensitivity analysis (per-protocol basis) was performed to compare the variability of results between filled and unfilled data. The χ^2^ test was used for the comparison of the PPCs incidence, presented as odds ratios (OR) and 95% confidence interval. The Mann–Whitney U test was used to compare the length of postoperative hospital stay and medical costs related to PPCs between the two groups. Subgroup analysis of primary and secondary outcomes was performed by different surgical methods. SPSS 25.0 (IBM Corp., Armonk, NY, USA) was used for all analyses, and we considered comparisons with *p*-values < 0.05 to be statistically significant.

## 3. Results

### 3.1 Participant Characteristics

**Fig. 1.**
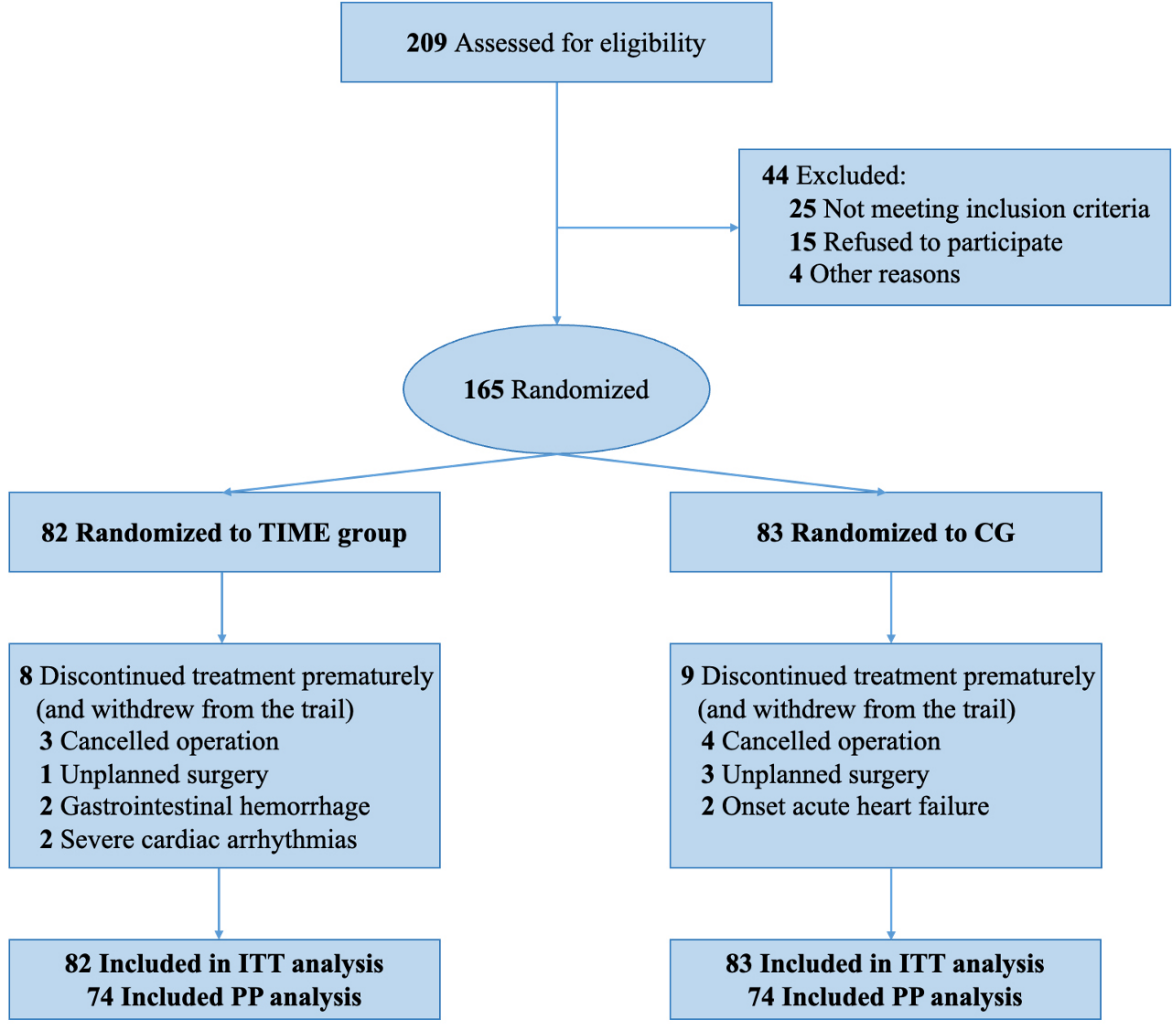
**Consolidated Standards of Reporting Trials (CONSORT) Diagram**. TIME, Three-day of Inspiratory muscle training, aerobic Muscle training, and Education; CG, control group; ITT, intention-to-treat; PP, per-protocol.

From August 2021 to September 2022, 209 patients awaiting elective valvular surgery were recruited by the Department of Cardiovascular Surgery at the West China Hospital, Sichuan University (Fig. 1). A total of 44 patients were excluded (25 for not meeting inclusion criteria, 15 for refusing to participate, and 4 for other reasons). 165 patients were randomly assigned to the two groups. 82 patients in the intervention (TIME) group and 83 in the control group provided data for the primary outcome.

Of 165 randomized patients, 53.94% were male, and the mean age was 63.41 years. The mean cardiovascular surgical risk score was 5.32, and the left ventricular ejection fraction (LVEF) was 56.64%. Baseline characteristics were shown in Table 1. The patient characteristics for both open-heart surgery and catheter-based interventions was shown in **Supplementary Table 1**.

**Table 1.** **Baseline demographics and clinical characteristics of the study population**.

		TIME (n = 82)	CG (n = 83)	*p* value
Age, mean (SD), y		63.48 ± 10.90	63.34 ± 9.61	0.591
Female, n (%)		37 (45.10)	39 (46.99)	0.810
Height, mean (SD), cm		161.09 ± 8.28	160.25 ± 7.46	0.498
Weight, mean (SD), kg		62.28 ± 10.88	60.07 ± 9.59	0.167
BMI, mean (SD), kg/$m^{2}$		23.98 ± 3.67	23.35 ± 3.28	0.251
History of smoke				0.241
	No smoking, n (%)	54 (65.90)	44 (53.00)	
	Cessation of smoking, n (%)	22 (26.80)	30 (36.10)	
	Smoking, n (%)	6 (7.30)	9 (10.80)	
NYHA classification				0.010
	II, n (%)	45 (54.90)	29 (34.90)	
	III, n (%)	37 (45.10)	54 (65.10)	
Euro Score, mean (SD)		5.22 ± 3.04	5.42 ± 3.48	0.692
LVEF, mean (SD), %		57.55 ± 11.11	55.73 ± 14.68	0.372
KCCQ, mean (SD)		62.06 ± 12.85	60.04 ± 13.88	0.335
Pulmonary symptoms				
	Cough, n (%)	36 (43.90)	27 (32.50)	0.133
	Expectoration, n (%)	19 (23.20)	23 (27.70)	0.503
	Wheezing, n (%)	2 (2.40)	9 (10.80)	0.030
	Dyspnea, n (%)	3 (3.70)	8 (9.60)	0.124
	Bronchial medication, n (%)	0 (0)	6 (7.20)	0.013
Comorbidities				
	Hypertension, n (%)	37 (45.10)	34 (41.00)	0.590
	Chronic obstructive pulmonary diseases, n (%)	25 (30.5)	34 (41.00)	0.160
	Asthma, n (%)	0 (0)	0 (0)	-
	Sleep apnea, n (%)	0 (0)	0 (0)	-
	Inspiratory muscle weakness, n (%)	53 (64.60)	56 (67.50)	0.701
	Coronary heart disease, n (%)	12 (14.60)	19 (22.9)	0.175
	Respiratory infection in the last month, n (%)	4 (4.90)	7 (8.40)	0.360
	Diabetes mellitus, n (%)	8 (9.80)	14 (16.90)	0.179
	Neurological disorders, n (%)	13 (15.90)	12 (14.5)	0.803
	History of median sternotomy, n (%)	5 (6.76)	5 (6.76)	0.57
Surgical approach				0.486
	TAVR, n (%)	46 (56.10)	51 (61.40)	
	Open-heart surgery, n (%)	36 (43.90)	32 (38.60)	

Data are expressed as number (%) and mean (SD). NYHA, New York Heart Association; LVEF, left ventricular ejection fraction; KCCQ, Kansas City Cardiomyopathy Questionnaire; TAVR, transcatheter aortic valve replacement; CG, control group; TIME, Three-day of Inspiratory muscle training, aerobic Muscle training, and Education; BMI, body mass index. For comparisons between groups at baseline, Chi square test, Student’s *t*-test and Mann-Whitney U test were used.

### 3.2 Primary Outcome

Overall, 70 of the 165 patients (42.42%) developed a PPCs. 26 (31.71%) patients in the TIME group and 44 (53.01%) patients in the control group developed a PPCs grade of at least 2. The difference between the two groups was statistically significant (OR, 0.60; 95% CI, 0.41–0.87, *p* = 0.006). The incidence of pneumonia in the TIME group was 11 (13.41%), which was statistically different compared to the control group with 35 (42.17%) (OR, 0.32; 95% CI, 0.17–0.58, *p* < 0.001). The incidence of pleural effusion in the TIME group was also significantly different compared with the control group (OR, 0.32; 95% CI, 0.16–0.68, *p* = 0.001) (Table 2). In the per-protocol analysis, the incedence of PPCs was also statistically significant between the two groups (OR, 0.60; 95% CI, 0.41–0.89, *p* = 0.008), as shown in **Supplementary Table 2**.

**Table 2.** **Characteristics of patients with postoperative pulmonary complications level on intention-to-treat basis**.

		TIME (n = 82)	CG (n = 83)	OR (95% CI)	*p* value
Level of PPCs					
	Grade 1	18 (21.95)	18 (21.69)	1.01 (0.57–1.80)	0.967
	Grade 2	53 (64.6)	39 (47.00)	1.38 (1.04–1.82)	0.022
	Grade 3	8 (9.80)	20 (24.1)	0.41 (0.19–0.87)	0.014
	Grade 4	3 (3.70)	6 (7.20)	0.51 (0.13–1.96)	0.313
^ε^PPCs (+)		26 (31.71)	44 (53.01)	0.60 (0.41–0.87)	0.006
Pleural effusion		8 (9.80)	25 (30.10)	0.32 (0.16–0.68)	0.001
Ventilation failure		2 (2.40)	6 (7.20)	0.34 (0.07–1.62)	0.152
Pneumonia		11 (13.41)	35 (42.17)	0.32 (0.17–0.58)	<0.001

Data are expressed as number (%) and OR (95% CI). CG, control group; PPCs, postoperative pulmonary complications; OR, odds ratio; TIME, Three-day of Inspiratory muscle training, aerobic Muscle training, and Education. Calculated using the Pearson χ^2^ or Fisher exact test; ε: Patients with 2 or more items in the Grade 2 complications or 1 item in Grade 3/4 complications.

In subgroup analyses, the two groups of patients undergoing open-heart surgery were similar in the incidence of PPCs (OR, 0.73; 95% CI, 0.49–1.09, *p* = 0.117). However, the incidence of pneumonia in the TIME group was statistically different compared to the control group (OR, 0.44; 95% CI, 0.23–0.85, *p* = 0.009) (Table 3). The results of the two groups of patients undergoing catheter-based intervention showed the lowest incidence of PPCs in the TIME group, which was statistically significant compared with the control group (OR, 0.40; 95% CI, 0.20–0.82, *p* = 0.006) (Table 4).

**Table 3.** **Characteristics of patients with postoperative pulmonary complications level in open-heart surgery population on intention-to-treat basis**.

		TIME (n = 36)	CG (n = 32)	OR (95% CI)	*p* value
Level of PPCs					
	Grade 1	3 (8.30)	3 (9.40)	0.89 (0.19–4.10)	0.880
	Grade 2	24 (66.70)	14 (43.8)	1.52 (0.97–2.40)	0.057
	Grade 3	6 (16.70)	9 (28.1)	0.59 (0.24–1.48)	0.255
	Grade 4	3 (8.30)	6 (18.80)	0.44 (0.12–1.63)	0.206
^ε^PPCs (+)		18 (50.00)	22 (68.80)	0.73 (0.49–1.09)	0.117
Pleural effusion		6 (16.70)	14 (43.80)	0.38 (0.17–0.87)	0.014
Ventilation failure		2 (5.60)	6 (18.8)	0.30 (0.06–1.37)	0.092
Pneumonia		9 (25.00)	18 (56.30)	0.44 (0.23–0.85)	0.009

Data are expressed as number (%) and OR (95% CI). CG, control group; PPCs, postoperative pulmonary complications; OR, odds ratio; TIME, Three-day of Inspiratory muscle training, aerobic Muscle training, and Education. Calculated using the Pearson χ^2^ or Fisher exact test; ε: Patients with 2 or more items in the Grade 2 complications or 1 item in Grade 3/4 complications.

**Table 4.** **Characteristics of patients with postoperative pulmonary complications level in catheter-based intervention population on intention-to-treat basis**.

		TIME (n = 46)	CG (n = 51)	OR (95% CI)	*p* value
Level of PPCs					
	Grade 1	15 (32.61)	15 (29.41)	1.11 (0.61–2.01)	0.734
	Grade 2	29 (63.04)	25 (49.00)	1.29 (0.90–1.84)	0.165
	Grade 3	2 (4.35)	11 (21.60)	0.20 (0.05–0.86)	0.013
	Grade 4	0 (0.00)	0 (0.00)	\	\
^ε^PPCs (+)		8 (17.40)	22 (43.10)	0.40 (0.20–0.82)	0.006
Pleural effusion		2 (4.30)	11 (21.60)	0.20 (0.05–0.86)	0.013
Ventilation failure		0 (0.00)	0 (0.00)	\	\
Pneumonia		2 (4.30)	17 (33.3)	0.13 (0.03–0.53)	<0.001

Data are expressed as number (%) and OR (95% CI). CG, control group; PPCs, postoperative pulmonary complications; OR, odds ratio; TIME, Three-day of Inspiratory muscle training, aerobic Muscle training, and Education. Calculated using the Pearson χ^2^ or Fisher exact test; ε: Patients with 2 or more items in the Grade 2 complications or 1 item in Grade 3/4 complications.

### 3.3 Secondary Outcomes

#### 3.3.1 Feasibility

Of 82 patients, 78 completed the pre-rehabilitation bundle as planned. Three patients canceled the operation and one patient brought forward the date of surgery. A total of 960 sessions of the bundle were conducted. No serious cardiovascular respiratory or other safety events occurred during the study period. Only two patients reported chest muscle soreness after IMT.

All participants in the TIME group returned the satisfaction questionnaire. The results showed the mean (SD) scores for satisfaction and motivation on a 10-point scale were 9.1 ± 0.8 and 8.6 ± 1.4, respectively (**Supplementary File 3**).

#### 3.3.2 Hospitalization and PPCs-Related Medical Costs

Patients in the TIME group had a shorter time on non-invasive mechanical ventilation compared to the control group after extubation (0.00 *vs*. 15.50 h, *p* = 0.043). However, the length of stay in the intensive care unit (2.00 *vs*. 2.00 d, *p* = 0.098) and in the postoperative stay (7.00 *vs*. 7.00 d, *p* = 0.110) between two groups were non-significant. Compared to the control group, patients in the TIME group spent less on PPCs-related medical costs (6.96 *vs*. 9.57 thousand CNY (1.01 *vs*. 1.39 thousand USD), *p* < 0.001) (Table 5). In the per-protocol analysis, patients in the TIME group also had a shorter time on non-invasive mechanical ventilation (0.00 *vs*. 16.00 h, *p* = 0.026), intensive care unit (2.00 *vs*. 2.00 d, *p* = 0.038), postoperative stay (7.00 *vs*. 7.00 d, *p* = 0.028) and less PPCs-related medical costs(6.88 *vs*. 9.64 thousand CNY (1.00 *vs.* 1.40 thousand USD), *p* < 0.001) (**Supplementary Table 3**).

**Table 5.** **Hospitalization and PPCs-related medical costs on intention-to-treat basis**.

	TIME (n = 82)	CG (n = 83)	*p* value
Duration of MV (h)	0.00 (0.00, 15.00)	0.00 (0.00, 14.45)	0.811
Duration of NIV (h)	0.00 (0.00, 22.00)	15.50 (0.00, 37.75)	0.043
Duration of ICU (d)	2.00 (1.00, 3.00)	2.00 (1.00, 3.00)	0.098
Postoperative stay (d)	7.00 (6.00, 9.00)	7.00 (6.00, 9.00)	0.110
PPCs-related cost (thousand, CNY)	6.96 (5.39, 8.08)	9.57 (8.08, 11.58)	<0.001
PPCs-related cost (thousand, USD)	1.01 (0.78, 1.17)	1.39 (1.17, 1.68)	<0.001

Data are expressed as median (inter quartile range). CG, control group; PPCs, postoperative pulmonary complications; MV, mechanical ventilation; NIV, Noninvasive mechanical ventilation; ICU, intensive care unit; CNY, China Yuan; TIME, Three-day of Inspiratory muscle training, aerobic Muscle training, and Education.

Subgroup analysis showed that the TIME group with catheter-based intervention had a shorter time on non-invasive ventilation after extubation (0 *vs*. 4.92 h, *p* = 0.005) and lower PPCs-related medical costs compared with the control group (6.83 *vs*. 9.27 thousand CNY (0.99 *vs.* 1.35 thousand USD), *p* < 0.001). The length of stay in the intensive care unit (1.00 *vs*. 1.00 d, *p* = 0.154) and postoperative stay (6.00 *vs*. 7.00 d, *p* = 0.103) between two groups were non-significant. Among open-heart surgery patients, the length of stay in the intensive care unit was shorter (2.00 *vs*. 3.00 d, *p* = 0.028) and have lower PPCs-related medical costs (7.05 *vs*. 9.69 thousand CNY (1.02 *vs.* 1.41 thousand USD, *p* < 0.001) in the TIME group compared with the control group. The duration of MV (15.00 *vs*. 15.98 h, *p *= 0.376), non-invasive ventilation (21.00 *vs*. 27.25 h, *p* = 0.401), and length of stay in the postoperative stay (7.00 *vs*. 7.00 h, *p* = 0.587) were shorter and the additional cost was less in the TIME group, however, the difference was not statistically significant (Tables 6,7).

**Table 6.** **Hospitalization and PPCs-related medical costs in catheter-based intervention population on intention-to-treat basis**.

	TIME (n = 46)	CG (n = 51)	*p* value
Duration of MV (h)	0.00 (0.00, 0.00)	0.00 (0.00, 0.00)	0.803
Duration of NIV (h)	0.00 (0.00, 8.25)	4.92 (0.00, 20.67)	0.005
Duration of ICU (d)	1.00 (1.00, 2.00)	1.00 (1.00–2.00)	0.154
Postoperative stay (d)	6.00 (5.75, 8.00)	7.00 (6.00, 9.00)	0.103
Total cost in respiratory (thousand, CNY)	6.83 (5.45, 7.94)	9.27 (7.64, 11.58)	<0.001
Total cost in respiratory (thousand, USD)	0.99 (0.79, 1.15)	1.35 (1.11, 1.68)	<0.001

Data are expressed as median (inter quartile range). CG, control group; PPCs, postoperative pulmonary complications; MV, mechanical ventilation; NIV, Noninvasive mechanical ventilation; ICU, intensive care unit; CNY, China Yuan; TIME, Three-day of Inspiratory muscle training, aerobic Muscle training, and Education.

**Table 7.** **Hospitalization and PPCs-related medical costs in open-heart surgery population on intention-to-treat basis**.

	TIME (n = 36)	CG (n = 32)	*p* value
Duration of MV (h)	15.00 (10.19, 18.98)	15.98 (12.03, 18.58)	0.376
Duration of NIV (h)	21.00 (4.44, 38.75)	27.25 (8.01, 46.48)	0.401
Duration of ICU (d)	2.00 (2.00, 4.00)	3.00 (3.00–4.00)	0.028
Postoperative stay (d)	7.00 (6.00, 10.00)	7.00 (7.00, 9.00)	0.587
Total cost in respiratory (thousand, CNY)	7.05 (5.10, 9.61)	9.69 (8.82, 11.58)	<0.001
Total cost in respiratory (thousand, USD)	1.02 (0.74, 1.40)	1.41 (1.28, 1.68)	<0.001

Data are expressed as median (inter quartile range). CG, control group; PPCs, postoperative pulmonary complications; MV, mechanical ventilation; NIV, Noninvasive mechanical ventilation; ICU, intensive care unit; CNY, China Yuan; TIME, Three-day of Inspiratory muscle training, aerobic Muscle training, and Education.

## 4. Discussion

We present for the first time a real-world feasibility and accessibility pre-rehabilitation bundle adapted to medical care in China. This bundle achieves healthy equality and the goal of reducing the incidence of PPCs, the duration of ventilation, and saving the related medical costs with limited resources.

In contrast to noncardiac surgery, there is still a lack of standardized pre-rehabilitation clinical pathways prior to elective cardiac surgery. One important reason for this may be that VHD patients are at high risk for exercise-induced adverse events. However, pre-rehabilitation is needed in patients with valve disease due to the chronic lack of physical activity and poor physical status [32]. There were no serious adverse events in this trial, except for two patients who complained of muscle soreness after the training. This indicates that with appropriate planning and supervision during the training session, the safety of the pre-rehabilitation bundle is acceptable. In addition, the satisfaction and completion results also suggested that patients were satisfied with the protocol. In China, patients prefer public hospitals [33] where resources are concentrated and therefore give more trust and patience to the medical service, which we believe is an important reason for the high level of satisfaction and compliance with the pre-rehabilitation bundle.

Heart valve surgery is a complex and high-risk procedure that presents significant physiological stress to the patient’s physical condition, particularly at the pulmonary level [3]. Almost all patients experience varying degrees of postoperative respiratory dysfunction after cardiac surgery [34], which has become a major cause of increased mortality and hospitalization costs [35, 36]. The disease progression of VHD requiring surgical intervention differs from that of coronary artery disease in that the early manifestations are symptoms of heart failure dominated by dyspnea as well as decreased exercise capacity. Increased cardiac load causes pulmonary congestion and possible alterations in pulmonary vascular function and structure, while increased respiratory muscle atrophy and fatigue combine to promote the progression of PPCs [37, 38].

Optimization of the patient’s preoperative health status, or “pre-rehabilitation”, should be a cornerstone of improved perioperative management to enhance preoperative physiological reserve [39]. The accumulation of evidence suggests that pre-rehabilitation was associated with a significantly reduced relative risk of developing PPCs (risk ratio (RR), 0.39, 95% CI 0.23–0.66) [31]. These physiotherapy interventions include preoperative respiratory muscle training, preoperative PT-led education, and exercise training, which were employed in our pre-rehabilitation bundle. In this trial, the results also found a 40.0% reduction in the overall incidence of PPCs in the TIME group, which is largely consistent with the previous results. Previous studies have shown that the combination of both physical training and IMT resulted in a PPCs reduction by at least 40%, with an average reduction of 60% PPCs [40]. This multimodal pre-rehabilitation program also included physiotherapy-led education, which is considered to be one of the most important components in reducing PPCs [8]. We believe that the effectiveness of the TIME pre-rehabilitation bundle may be attributed to its ability to maximize postoperative oxygen transport in patients within a short preoperative period. Patients are taught to perform unloaded breathing exercises and effective coughing in an intensive preoperative education program to better understand and apply these techniques to avoid sputum retention and lobar lung collapse in the early postoperative period [41]. Preoperative exercises such as thoracic stretching may also help patients increase thoracic compliance to reduce respiratory work. Furthermore, short-term preoperative loaded IMT increases respiratory (muscle) function, which may counteract postoperative weakening of inspiratory muscles caused by anesthesia and pain [42]. Finally, walking training based on a 6-minute walk test allows patients to remain active preoperatively. All of these measures optimize patient oxygen transport, which may be responsible for reducing the risk of PPCs and the resulting reduction in length of stay and medical cost.

Importantly, the differences were found in the benefit of two types of surgical populations the in same program. This pre-rehabilitation bundle did not appear to change the incidence of PPCs in patients undergoing open-heart surgery. The possible explanation is that procedure-related risk, especially the surgical site, was found to be the most important predictor of risk for PPCs in cardiac surgery [43]. In addition, the mechanical injury traction imposed by median sternotomy, the establishment of extracorporeal circulation, saline cryopreservation due to hypothermic myocardial protection, and rapid shallow breathing patterns due to postoperative sedation for pain all negatively affect pulmonary function [44, 45, 46]. Therefore, a short-term (three-day) preoperative pre-rehabilitation bundle may not be enough for the patient to gain sufficient physiological reserve to overcome the effects of open-heart surgery. Catheter-based intervention such as transcatheter aortic valve replacement (TAVR) is an alternative option for patients with multiple comorbidities and high perioperative mortality for open-heart surgery because it is less invasive and avoids the need for cardiopulmonary bypass [47]. Thus, adequate preoperative preparation may also be important for this high-risk group of patients undergoing catheter-based intervention. Our study confirms that even a short-term pre-rehabilitation bundle can benefit catheter-based intervention patients. However, subgroup analysis is influenced by sample size, so this will need to be explored in future adequately powered studies.

The presence of PPCs is accompanied by a series of high-cost events and is a major cause of prolonged ventilation support and length of stay in the intensive care unit [48]. Even mild PPCs were associated with increased healthcare resource utilization. Our findings showed a shorter duration of non-invasive ventilatory support. However, there were still conflicting findings about the impact of pre-rehabilitation on the length of stay based on previous studies [13]. The length of hospital stay and the choice of time and mode of ventilatory support for patients in clinical practice are influenced by multiple factors. Therefore, the independent effect of PPCs on length of stay may be smaller than previously reported when confounding factors are considered [8]. However, the situation may be different in Chinese VHD patients with valve disease, as these patients may already be at a low preoperative baseline level due to a chronic lack of adequate health care, and thus the effect of pre-rehabilitation may be more pronounced in this group. These are topics that need to be explored in future research.

As the population ages and cost pressures increase on the healthcare system, the cost-effectiveness of CR is an important topic. If CR does not achieve a high level of cost-effectiveness for patients, it will be difficult to obtain government support. The results of this study demonstrated fewer additional medical costs resulting from PPCs (6.96 *vs*. 9.57 thousand CNY (1.01 vs. 1.39 thousand USD)) in patients who received the pre-rehabilitation program. The medical cost of pre-rehabilitation is comparatively affordable compared to the cost of treating a PPCs in hospital [27]. According to the National Bureau of Statistics of China, in 2021, the per capita disposable income of Chinese residents is 2.93 thousand CNY (0.43 thousand USD) per month. This means that the pre-rehabilitation bundle can save almost a month of the population’s disposable income in China, which can be very important in relieving patients’ financial stress. In addition, according to the current inpatient medical reimbursement policy, the cost of three days of pre-rehabilitation in a public hospital for 600 CNY (87.20 thousand USD) is affordable for the patient, which is one of the main reasons why the pre-rehabilitation bundle is acceptable. Overall, this pre-rehabilitation bundle is significantly different from the previous pre-rehabilitation programs in that we have changed the place and time of delivery to a cardiac surgery unit with pre-operative waiting times. Lack of community-based rehabilitation resources, remote populations that are not easily reached, and health insurance that does not support out-of-hospital rehabilitation are all common dilemmas that most resource-limited countries need to face. Our proposed pre-rehabilitation bundle provides a practical solution to this dilemma and promotes medical equity in CR delivery.

## 5. Limitations

The study has several limitations. In this randomized controlled trial, due to resource constraints, both groups were assessed and trained by the same physical therapist team. In daily clinical practice, there may be large differences in the therapist’s experience as well as in his or her manipulative capabilities, and the results obtained may vary widely. Therefore, it is essential to study the pre-rehabilitation bundle in clinical practice in cardiac centers of different sizes [49]. In addition, the trial results should be interpreted with more caution because some patient data may be missing during the implementation process. However, we imposed a sensitivity analysis and judged that the filled data interfered little with the interpretation of the trial results. Moreover, although the randomization groups showed comparable baseline population characteristics, our study’s most significant influencing factor, the surgical procedure, had a significant effect on outcome events. Sensitivity analyses assessed the effect of the intervention bundle on populations with different surgical approaches. Previous studies have focused on patients undergoing coronary artery bypass grafting and heart valvular surgery with open-heart surgery [50]. Future studies should focus on specific types of surgery rather than all cardiac patients to help provide a more accurate understanding of the role of this intervention in specific populations. Finally, perioperative management of patients undergoing cardiac surgery should include both preoperative and postoperative components, whereas this study only explored in-hospital pre-rehabilitation protocols. The perioperative and post-discharge health management of cardiac surgery patients with current limited resources remains an issue that needs to be explored in the future. We continue to believe that home and community-based pre-rehabilitation will remain the mainstream in the future but is not currently available in developing countries. With the advancement of digital therapies [51], AI-based home pre-rehabilitation programs offer a good solution to the lack of medical resources in developing countries.

## 6. Conclusions

This trial explored a three-day accessible pre-rehabilitation bundle. It minimizes the incidence of PPCs and length of hospital stay in patients undergoing elective cardiac surgery through multidisciplinary collaboration with limited resources and maximizes the healthy equity of CR. This study also demonstrates that short-term preoperative pre-rehabilitation can save medical costs and provide an affordable model for the expansion of pre-rehabilitation in LMICs.

## Data Availability

The datasets used and/or analyzed during the current study are available from the corresponding author on reasonable request.

## Supplementary Material

Supplementary material associated with this article can be found, in the online version, at https://doi.org/10.31083/j.rcm2411308.
