# Supplementary material for: An Accessible Pre-Rehabilitation Bundle for Patients Undergoing Elective Heart Valve Surgery with Limited Resources: The TIME Randomized Clinical Trial
Source: Rev Cardiovasc Med. 2023 Nov 9;24(11):308. doi: 10.31083/j.rcm2411308 (PMC11272831; doi:10.31083/j.rcm2411308)
Supplement: Supplementary file 1 [file 2153-8174-24-11-308-s1.docx]

**Supplementary File 1: Preoperative education information protocol (by a physiotherapist).**

**Information Part**

- Knowledge of PPCs and other common complications after valvular surgery
- Principles of deep breathing and effective coughing
- Introduction to the importance of early mobilization
- Introduction to the work of physiotherapists in intensive care unit

**Education and practice part**

- Respiratory training

- Diaphragmatic breathing, diaphragm muscles could be strengthened through this exercise (assignment after training session:5sets/day,15 repetitions each set following 2-3 min rest for three sets).

- Effective coughing techniques (assignment after training session:5sets/day,15 repetitions each set following 2-3 min rest for three sets)

- Self-stretching technique

- Self thoracic stretching (assignment after training session:5sets/day,15 repetitions each set following 2-3 min rest for three sets)

**-** Self extremities stretching (assignment after training session:5sets/day,15 repetitions each set following 2-3 min rest for three sets)

- Early mobilization tips

-Bed exercise: ankle pump, leg lift, extremity isometric contraction, fist clenching, cranking exercise

-Positioning and transfer technique in intensive care unit

-Postoperative sternal protection tips for patients undergoing median sternotomy.

- In each education session, the PT will ask the patient to repeat the contents to ensure subjects have mastered these techniques. In addition, the PT will answer questions about the patient's cardiac surgery and provide additional information based on the patient's occupation and life situation.

**Supplementary File 2: Security Event Determination Criteria.**

| **Cardiovascular related events** | **Respiratory related events** | **Neurological related events** | **Other medical related Events** |
| --- | --- | --- | --- |
| - Angina/chest pain (ECG ischemic changes) - Severe cardiac arrhythmias (hemodynamic instability) - Cyanosis - Hypotension (MAP <60mmHg or SBP <90mmHg for more than 2min) - Hypertension (MAP>140mmHg or SBP>180mmHg for more than 2min) - Bradycardia (HR <50bpm and lasting more than 2min) - Tachycardia (HR > 140 bpm for more than 2 min) - Cardiac arrest | - Dyspnea - Assisted respiratory muscle use - Reduced oxygen saturation: SpO_2_ <85% for more than 1min without oxygen; SpO_2_ <90% for more than 1min with oxygen - Excessive respiratory rate (RR > 30 bpm for more than 2 min). | - Syncope or loss of consciousness | - Adverse events considered by clinicians but not listed |

Abbreviation: MAP, mean arterial pressure; SBP, systolic blood pressure; HR, heart rate.

**Supplementary File 3: Patient evaluation of the pre-rehabilitation program.**

1) What is your overall opinion of the pre-rehabilitation program?

Fairly good  ***0(0%)***

Good  ***29(39%)***

Very good  ***45(61%)***

2) How would you qualify the intensity about the pre-rehabilitation program?

Light ***0(0%)***

Ideal ***62(84%)***

Heavy ***12(16%)***

3) How would you qualify the supervision given by the physical therapist and cardiac nurse?

Not so good  ***0(0%)***

Good  ***4(5%)***

Very good ***70(95%)***

4) How motivated were you during the pre-rehabilitation program?

Considered quitting  ***0(0%)***

Motivated ***21(28%)***

Very motivated ***53(72%)***

5) Did you experience the effect of the pre-rehabilitation program?

Yes ***52(70%)***

I don’t know ***20(27%)***

No ***2(3%)***

6) How would you qualify the overall organization?

Bad ***0(0%)***

Good ***21(28%)***

Very good ***53(72%)***

7) Would you participate again in a pre-rehabilitation program?

Yes ***70(96%)***

No ***4(4%)***

8) How would you rate the pre-rehabilitation program on a scale from 1 to 10 (10/very bad, 100/excellent)? (mean±SD)  ***9.3(±0.8)***

9) How would you rate your satisfaction of the pre-rehabilitation program on a scale from 1 to 10 (10/very bad, 100/excellent)? (mean±SD)  ***9.1(±0.8)***

10) How would you rate your motivation for pre-rehabilitation program on a scale from 1 to 10 (10/very bad, 100/excellent)? (mean±SD) ***8.6(±1.4)***

**Supplementary Table 1. Demographic and Clinical Characteristics in different intervention approaches.**

|  | **Catheter-based intervention (n=97)** | **Open-heart surgery**  **(n=68)** | ***p* value** |
| --- | --- | --- | --- |
| **Age, mean (SD), y** | 68.37 ± 7.08 | 57.54 ± 10.75 | <0.001 |
| **Female** | 46 (47.40) | 30 (44.10) | 0.675 |
| **Height, mean (SD), cm** | 160.07 ± 8.40 | 161.51 ± 7.00 | 0.247 |
| **Weight, mean (SD), kg** | 60.62 ± 11.25 | 61.94 ± 8.74 | 0.399 |
| **BMI, mean (SD), kg/m^2^** | 23.61 ± 3.80 | 23.74 ± 2.99 | 0.807 |
| **History of smoke** |  |  | 0.869 |
| No smoking, n (%) | 56 (57.70) | 42 (61.80) |  |
| Cessation of smoking, n (%) | 32 (33.00) | 20 (29.40) |  |
| Smoking, n (%) | 9 (9.30) | 6 (8.80) |  |
| **NYHA classification** |  |  | 0.152 |
| II, n (%) | 39 (40.20) | 35 (51.50) |  |
| III, n (%) | 58 (59.80) | 33 (48.50) |  |
| **Euro Score, mean (SD)** | 6.56 ± 3.18 | 3.46 ± 2.48 | <0.001 |
| **LVEF, mean (SD), %** | 54.63 ± 14.61 | 59.50 ± 9.75 | 0.018 |
| **KCCQ, mean (SD)** | 58.11 ± 13.31 | 65.23 ± 12.42 | 0.001 |
| **Pulmonary symptoms** |  |  |  |
| Cough, n (%) | 38 (39.20) | 25 (36.80) | 0.754 |
| Expectoration, n (%) | 26 (26.80) | 16 (23.50) | 0.635 |
| Wheezing, n (%) | 7 (7.20) | 4 (5.90) | 0.735 |
| Dyspnea, n (%) | 8 (8.20) | 3 (4.40) | 0.331 |
| Bronchial medication, n (%) | 6 (6.20) | 0 (0) | 0.037 |
| **Comorbidities** |  |  |  |
| Hypertension, n (%) | 48 (49.50) | 23 (33.80) | 0.046^*^ |
| Chronic obstructive pulmonary diseases, n (%) | 55 (56.70) | 4 (5.90) | <0.001^*^ |
| Asthma, n (%) | 0 (0) | 0 (0) | - |
| Sleep apnea, n (%) | 0 (0) | 0 (0) | - |
| Inspiratory muscle weakness, n (%) | 68 (70.10) | 41 (60.30) | 0.190 |
| Coronary heart disease, n (%) | 30 (30.90) | 1 (1.50) | <0.001 |
| Respiratory infection in the last month, n (%) | 5 (5.20) | 6 (8.80) | 0.352 |
| Diabetes mellitus, n (%) | 15 (15.50) | 7 (10.30) | 0.336 |
| Neurological disorders, n (%) | 20 (20.60) | 5 (7.40) | 0.019^*^ |
| History of median sternotomy, n (%) | 6 (6.20) | 4 (5.9) | 0.936 |
| **The surgical approach** |  |  |  |
| TAVR, n (%) | 97(100) |  | - |
| AVR, n (%) | - | 18(26.47) | - |
| MVR, n (%) | - | 9(13.24) | - |
| AVR+TVP, n (%) | - | 6(8.82) | - |
| MVR+TVP, n (%) | - | 24(35.29) | - |
| AVR+MVR+TVP, n (%) | - | 11(16.18) | - |

Data are expressed as mean ± SD, number (%). BMI, Body mass index; NYHA, New York Heart Association; LVEF, left ventricular ejection fraction; KCCQ, Kansas City Cardiomyopathy Questionnaire; TAVR, Transcatheter aortic valve replacement; AVR, Aortic valve replacement; MVR, Mitral valve replacement; TVP, tricuspid valve plasty.

**Supplementary Table 2. Characteristics of patients with postoperative pulmonary complications on per-protocol basis.**

|  | **TIME (n=74)** | **CG (n=74)** | **OR (95%CI)** | ***p* value** |
| --- | --- | --- | --- | --- |
| **Level of PPCs** |  |  |  |  |
| **Grade 1** | 14 (18.90) | 14 (18.9) | 1.00 (0.51-1.95) | 1.000 |
| **Grade 2** | 50 (67.57) | 37 (50.00) | 1.35 (1.02-1.78) | 0.030 |
| **Grade 3** | 8 (10.81) | 18 (24.32) | 0.44 (0.21-0.96) | 0.031 |
| **Grade 4** | 2 (2.70) | 5 (6.76) | 0.40 (0.08-2.00) | 0.245 |
| **^ε^ PPCs (+)** | 24 (32.40) | 40 (54.10) | 0.60 (0.41-0.89) | 0.008 |
| **Pleural Effusion** | 8 (10.81) | 22 (29.73) | 0.36 (0.17-0.76) | 0.004 |
| **Ventilation Failure** | 2 (2.70) | 5 (6.76) | 0.40 (0.08-2.00) | 0.245 |
| **Pneumonia** | 10 (13.51) | 31 (41.89) | 0.32 (0.17-0.61) | <0.001 |

Data are expressed as numbers (%) and OR (95%CI). TIME, Three-day of Inspiratory muscle training, aerobic Muscle training, and Education; CG, control group; PPCs, Postoperative pulmonary complications; OR, odds ratio. Calculated using the Pearson χ^2^ or Fisher exact test; ε: Patients with 2 or more items in the Grade 2 complications or 1 item in Grade 3/4 complications.

**Supplementary Table 3. Hospitalization and PPCs-related cost on per-protocol basis.**

|  | **TIME (n=74)** | **CG (n=74)** | ***p* value** |
| --- | --- | --- | --- |
| **Duration of MV (h)** | 0.00 (0.00, 14.21) | 0.00 (0.00, 14.34) | 0.926 |
| **Duration of NIV (h)** | 0.00 (0.00, 21.44) | 16.00 (0.00, 40.25) | 0.026 |
| **Duration of ICU (d)** | 2.00 (1.00, 2.00) | 2.00 (1.00, 3.00) | 0.038 |
| **Postoperative stay (d)** | 7.00 (6.00, 9.00) | 7.00 (6.00, 9.00) | 0.028 |
| **PPCs-related cost (thousand, CNY)** | 6.88 (5.30, 7.94) | 9.64 (8.17, 12.29) | <0.001 |
| **PPCs-related cost (thousand, USD)** | 1.00 (0.77, 1.15) | 1.40 (1.19, 1.79) | <0.001 |

Data are expressed as median (inter quartile range). TIME, Three-day of Inspiratory muscle training, aerobic Muscle training, and Education; CG, control group; MV, mechanical ventilation; NIV, Noninvasive mechanical ventilation; ICU, intensive care unit; CNY, China Yuan.
